# Recurrent Otogenic Intracranial Sepsis: A Key Radiological Finding, Not to Be Missed

**DOI:** 10.1155/2019/5013932

**Published:** 2019-05-26

**Authors:** Mark Aziz, Eugene Omakobia

**Affiliations:** ENT Department, Hull and East Yorkshire Hospitals NHS Trust, Anlaby Road, Hull HU3 2JZ, UK

## Abstract

**Introduction:**

Otogenic intracranial sepsis is a well-known and established complication of otitis media. It is a major cause of morbidity and mortality from otitis media. We present a case of recurrent otogenic intracranial sepsis and key findings on imaging.

**Case Report:**

A 64-year-old male presented with two episodes of severe sepsis secondary to right sided otitis media. During the first episode, he suffered an episode of otogenic pneumococcal sepsis requiring intensive care admission. A year later, he suffered another episode of otogenic intracranial sepsis with evidence of encephalitis, which also required intensive care input. He underwent surgical management of his otitis media with a right myringotomy followed by grommet insertion.

**Findings:**

Both computerised tomography and magnetic resonance imaging of the temporal bones demonstrated a defect in the right tegmen tympani, through which a cyst herniated into the epitympanum. Postinfective changes were also noted in the right inferior temporal lobe.

**Discussion:**

Tegmen tympani defects are a rare but important risk factor for the spread of intracranial infections from the middle ear. In cases of recurrent otogenic intracranial sepsis, it is crucial to look for evidence of this finding on imaging.

## 1. Introduction

Otitis media (OM) is infection of the middle ear. OM commonly occurs in association with viral upper respiratory tract infections (URTIs), through dysfunction of the eustachian tube. Common organisms implicated in otitis media are *Streptococcus pneumoniae*, *Haemophilus influenzae*, and *Moraxella catarrhalis*, which are common pathogens found in URTIs. Presentation is usually with otalgia, hearing loss, and fever with subsequent purulent otorrhoea [1].

The reason that otitis media is important is due to the risks of associated complications. An acute episode of otitis media can progress to chronic otitis media or otitis media with effusion [1].

There is also a risk of spread of the infection. Infection can spread to the inner ear, causing labyrinthitis. Infection may also spread to the temporal bone causing mastoiditis. Unresolved otitis media can lead to intracranial extension of infection, leading to intracranial sepsis, manifesting itself as meningitis, brain abscess, or lateral sinus thrombosis [2, 3].

## 2. Case Report

We report the case of a 64-year-old man with previous history of multiple left sided childhood ear infections requiring multiple grommets. He also had a history of mixed hearing loss since 2015, which had been managed conservatively.

On 21^st^ February 2017, whilst on holiday, the patient suffered right sided otitis media (OM). The OM was complicated by pneumococcal sepsis, requiring ICU admission for observation and treatment. The patient was eventually discharged on a course of oral antibiotics. A computerised tomography (CT) scan of his head carried out during his admission was reported as normal. He was left with reduced hearing in the right ear due to right sided otitis media with effusion that resolved spontaneously on follow-up 6 months later.

The patient became unwell again while on holiday on 20^th^ April 2018. He presented to his local emergency department with confusion, photophobia, and agitation on the background of a 2-day history of right sided otalgia.

The CT head scan showed signs of right temporal lobe encephalitis and right middle ear opacification.

The patient underwent surgical management of the OM, with myringotomy, washout, and right sided grommet insertion on 20/04/2018. He was then treated with intravenous ceftriaxone and rifampicin but had to undergo sedation due to the severity of agitation.

He eventually settled on antibiotics and was discharged home from ICU for ENT follow-up at his local hospital.

## 3. Findings

**Figure 1:**
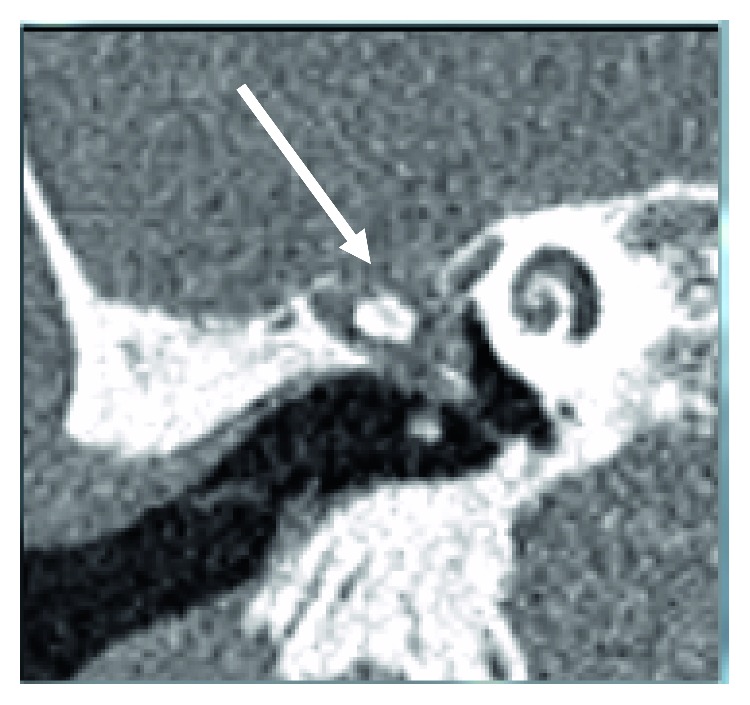
CT scan with the defect shown: high-resolution CT petrous bones (coronal view).

A repeat CT scan of his head carried out on 8^th^ July 2018, on follow-up with ENT, revealed a large defect in the right tegmen tympani (see Figure 1). The defect measured 6 × 7 mm in diameter. There was also an area of opacification in the epitympanum, which is likely due to a posttraumatic meningocele herniating through the defect.

**Figure 2:**
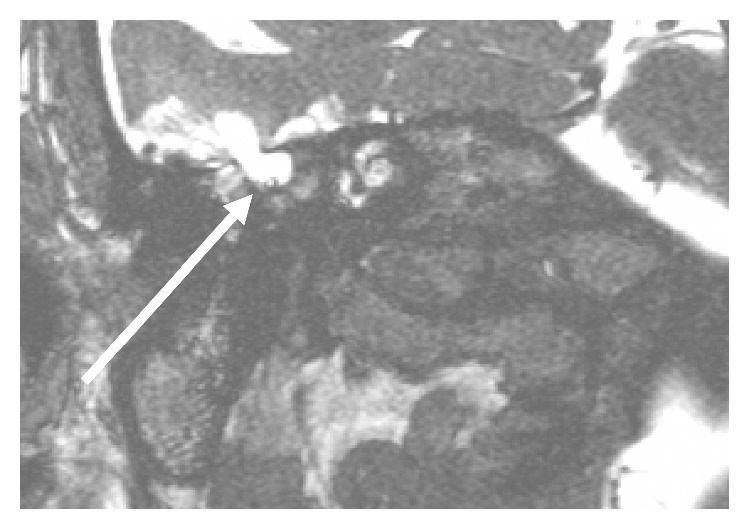
MRI scan with defect and the herniating cyst: MRI diffusion-weighted scan (coronal view).

These findings were again seen on an MRI scan carried out on 09/09/2018 to better visualize the herniating meningocele (see Figure 2).

## 4. Discussion

As mentioned in the introduction, OM can be complicated by intracranial spread of infection, and this is a well-known complication. In this case, the defect in the tegmen tympani provided a route for the spread of otogenic sepsis intracranially [4]. Tegmen tympani defects have been found on autopsy in 15–35% of patients who present with otorrhoea, suggesting they are not uncommon [5]. There are various theories postulating why these defects occur, with suggestions that this could be due to constant cerebrospinal fluid (CSF) pressure, CSF from aberrant arachnoid granulation tissue causing pressure on the bone, idiopathic intracranial hypertension, and morbid obesity [4]. This was likely the cause for the recurrent episodes of severe intracranial sepsis that our patient suffered as it is a well-known risk factor for intracranial infections [3].

A tegmen tympani defect is an isolated defect in the thin sheet of bone separating the cranial and tympanic cavity. This may be associated with herniation of intracranial contents and is described as a meningocele if dura alone or meningoencephalocele if dura and brain tissue. If there is a breach in the dura, there may also be a leak of CSF through the defect [4].

If a history of previous ear surgery, especially involving the mastoid, is present, then this should lead to consideration of the presence of a possible tegmen tympani defect. Imaging in such patients should take this into account.

This finding was picked up on follow-up scans. Had it been picked up earlier, it could have affected the management plan for subsequent episodes of OM, with low threshold for surgical management through myringotomy and grommet insertion, thereby avoiding the spread of sepsis. Moreover, early consideration of surgical repair of the tegmen defect should be considered to prevent further cases of intracranial spread of infection. Such surgery would usually require joint ENT and neurosurgical input.

There are 3 possible approaches for the repair of tegmen tympani defects discussed in the literature, and the choice depends on various factors including location and size of the defect [5]. The three approaches are transmastoid approach, middle fossa craniotomy approach, or the combined approach [5].

The transmastoid approach is a minimally invasive approach performed via a cortical mastoidectomy. The tegmen, mastoid, and sigmoid sinus are skeletonized. The herniating meningocele/encephalocele is excised and sent for histology. The repair may then be performed using shaped concha cartilage and temporalis fascia [4, 6].

The middle cranial fossa approach can be used for large/multiple defects and for anterior and medial site defects that are inaccessible via the transmastoid approach [5]. This approach usually involves making one or two burr holes to create a craniotomy. The dura is then elevated to expose the defects. Again the meningocele/encephalocele is identified and excised. The defect is repaired, with options for grafts including temporalis fascia, autologous bone/cartilage graft, or artificial grafts [5, 7].

The combined approach involves a wide mastoidectomy, skeletonizing the mastoid, tegmen, and sigmoid sinus. After identifying the defect, anterior epitympanotomy is performed for access. The encephalocele or meningocele is identified and amputated. Then, a minicraniotomy is performed and the dura elevated, to expose the tegmen defect intracranially. A repair is performed using single or multilayer techniques using a combination of temporalis fascia, fibrin glue, and artificial graft material such as Duragen graft. The advantage of this approach is that in patients with normal hearing, the ossicular chain and the patient's hearing can be preserved [8].

Aside from size and location, other factors involved in deciding on the appropriate approach include available grafts and the experience of the operating surgeon [5].

It would seem sensible to postulate that the larger the size of the defect, the greater the risk of meningocele/encephalocele formation. However, evidence for this could not be clearly identified in the literature.

In this case, our patient was discussed with an experienced neuroradiologist as well as with the local neurosurgical team. The outcome of that discussion was to take a conservative approach towards management as it was likely that the defect was sealed with scarring. A repeat MRI was repeated in February 2019, which revealed no changes. The patient was subsequently kept under regular surveillance in an outpatient setting.

## 5. Conclusion

Tegmen tympani defects are an important radiological sign, accounting for increased risk of spread of otogenic sepsis to the cranial cavity. This should be actively sought for in patients with otogenic intracranial sepsis, especially with recurrent episodes. The finding of a tegmen tympani defect should lead to reduced threshold for surgical management of otitis media. This consists of myringotomy and grommet insertion alongside intravenous antibiotics as the priority is to drain and treat the acute infection before consideration of formal repair of the defect once the acute infection has settled.
